# Overlapping Functions of the Paralogous Proteins AtPAP2 and AtPAP9 in *Arabidopsis thaliana*

**DOI:** 10.3390/ijms22147243

**Published:** 2021-07-06

**Authors:** Renshan Zhang, Xiaoqian Guan, Meijing Yang, Yee-Song Law, Chia Pao Voon, Junran Yan, Feng Sun, Boon Leong Lim

**Affiliations:** 1 School of Biological Sciences, The University of Hong Kong, Hong Kong, China; rs_zhang@fudan.edu.cn (R.Z.); sophia.xiaoqianguan@gmail.com (X.G.); celine30@connect.hku.hk (M.Y.); yeesong0210@gmail.com (Y.-S.L.); VoonChiaPao@hotmail.com (C.P.V.); judithayan@gmail.com (J.Y.); epusun@sdu.edu.cn (F.S.); 2 State Key Laboratory of Agrobiotechnology, The Chinese University of Hong Kong, Hong Kong, China

**Keywords:** AtPAP2, AtPAP9, chloroplasts, import, mitochondria, purple acid phosphatase

## Abstract

*Arabidopsis thaliana* purple acid phosphatase 2 (AtPAP2), which is anchored to the outer membranes of chloroplasts and mitochondria, affects carbon metabolism by modulating the import of some preproteins into chloroplasts and mitochondria. AtPAP9 bears a 72% amino acid sequence identity with AtPAP2, and both proteins carry a hydrophobic motif at their C-termini. Here, we show that AtPAP9 is a tail-anchored protein targeted to the outer membrane of chloroplasts. Yeast two-hybrid and bimolecular fluorescence complementation experiments demonstrated that both AtPAP9 and AtPAP2 bind to a small subunit of rubisco 1B (AtSSU1B) and a number of chloroplast proteins. Chloroplast import assays using [^35^S]-labeled AtSSU1B showed that like AtPAP2, AtPAP9 also plays a role in AtSSU1B import into chloroplasts. Based on these data, we propose that AtPAP9 and AtPAP2 perform overlapping roles in modulating the import of specific proteins into chloroplasts. Most plant genomes contain only one PAP-like sequence encoding a protein with a hydrophobic motif at the C-terminus. The presence of both *AtPAP2* and *AtPAP9* in the Arabidopsis genome may have arisen from genome duplication in Brassicaceae. Unlike AtPAP2 overexpression lines, the AtPAP9 overexpression lines did not exhibit early-bolting or high-seed-yield phenotypes. Their differential growth phenotypes could be due to the inability of AtPAP9 to be targeted to mitochondria, as the overexpression of AtPAP2 on mitochondria enhances the capacity of mitochondria to consume reducing equivalents.

## 1. Introduction

Purple acid phosphatases (PAPs) are present in yeasts, animals, and plants. These enzymes contain seven conserved metal-coordinating amino acid residues in five invariant blocks—DXG, GDXXY, GNH(D/E), VXXH, and GHXH [1,2]. The functional roles of plant purple acid phosphatases are diverse, including mobilization of internal and external phosphorus [3,4], cell wall generation [5], use of extracellular ATP [6], and regulation of stress responses [7]. Among the 29 purple acid phosphatases (AtPAPs) in the Arabidopsis genome, only AtPAP2 and AtPAP9 carry an extended hydrophobic C-terminal motif at their C-termini. AtPAP2 is targeted to the outer membranes of both plastids and mitochondria by this unique C-terminal hydrophobic motif [8,9] and plays a role in the import of some nucleus-encoded proteins into chloroplasts and mitochondria [10,11]. Many nucleus-encoded proteins imported into chloroplasts and mitochondria are targeted by their N-terminal transit peptides/presequences, which are recognized by receptor proteins in the translocon of the outer membranes of chloroplasts (TOC) and translocon of the outer membranes of mitochondria (TOM), respectively [12,13]. Some transit peptides/presequences are phosphorylated by cytosolic STY kinases [14,15], and the dephosphorylation of these phosphorylated transit peptides/presequences is required prior to the import of proteins into chloroplasts and mitochondria [16,17]. AtPAP2 is a phosphatase on the outer membranes of chloroplasts and mitochondria that recognizes these phosphorylated preproteins [10,11]. The overexpression of AtPAP2 on both organelles promotes the plant growth and seed yield of *Arabidopsis thaliana* by coordinating the physiology of chloroplasts and mitochondria simultaneously [8,18]. The chloroplasts of the AtPAP2 OE line exhibit a higher PSI/PSII ratio and ETR and fix more CO_2_, whereas the mitochondria of the AtPAP2 OE line can consume more reducing equivalents and generate more ATP compared with the WT line [19]. The higher consumption of reducing equivalents in the mitochondria can indirectly enhance the regeneration of stromal NADP^+^, electron acceptors of the linear electron flow [19], and thus the leaves of the AtPAP2 overexpression line contain higher ATP/NADPH and NADP^+^/NADPH ratios during photosynthesis compared with the WT line [18]. Surprisingly, when AtPAP2 is solely overexpressed in mitochondria, the transgenic lines exhibit high leaf-ATP, low sucrose, low seed-yield, and early-senescence phenotypes, possibly due to overreactive mitochondria [20].

AtPAP9 displays a 72% sequence similarity with AtPAP2, and both phosphatases carry hydrophobic C-terminal motifs [8,21]. Here, we show that AtPAP9 and AtPAP2 serve partially redundant biological functions in chloroplast protein import. However, unlike AtPAP2, overexpression of AtPAP9 in *Arabidopsis thaliana* does not promote plant growth, possibly due to its inability to be targeted to mitochondria and modulate mitochondrial activity [19]. 

## 2. Results

### 2.1. The C-Terminal Hydrophobic Motif of AtPAP9 Targets GFP to Chloroplasts

Previous studies have shown that AtPAP2 is dually targeted to plastids and mitochondria via its C-terminal hydrophobic motif [8,22]. The C-terminal hydrophobic motif of AtPAP9 (Figure S1) has a hydrophobicity value that is similar to that of AtPAP2 as well as that of the translocase of the outer chloroplast membrane (TOC) and the translocase of the outer mitochondrial membrane (TOM) (Table S1) [8]. Given the high sequence similarity (72% amino acid identity) between AtPAP2 and AtPAP9 (Figure S2), we investigated the targeting ability of the AtPAP9 C-terminal hydrophobic motif with green fluorescence protein (GFP) tagging. Forty-six amino acid residues at the C-terminus of AtPAP9 (a.a. 606–651) were fused to the C-terminus of GFP, and transient expression of the GFP fusion constructs was performed in Arabidopsis protoplasts. 

**Figure 1:**
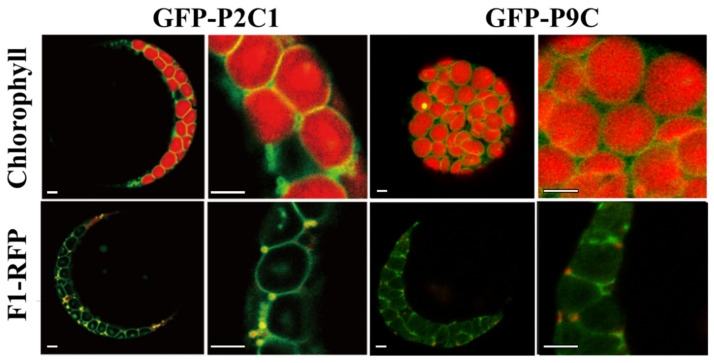
Targeting of GFP to organelles in Arabidopsis protoplasts by the C-terminal motifs of AtPAP2 and AtPAP9. GFP-P2C1 was targeted to the outer membranes of chloroplasts and mitochondria, and GFP-P9C was targeted to the outer membrane of only chloroplasts. Chlorophyll was the chloroplast marker, and F1-RFP was the mitochondrion marker. Scale bar = 5 μm.

Fluorescence microscopy analysis revealed that the GFP fused to the C-terminal hydrophobic motif of AtPAP9 (GFP-P9C) is targeted to the outer membrane of chloroplasts but does not co-localize with the mitochondrial marker. RFP fused with the mitochondrial ATP synthase subunit beta 1 (F1-RFP), whereas the C-terminal hydrophobic motif of AtPAP2 (GFP-P2C1) was targeted to the outer membranes of both chloroplasts and mitochondria (Figure 1). This indicated that the C-terminal hydrophobic motif of AtPAP9 targets GFP to chloroplasts but not to mitochondria. Although both C-terminal hydrophobic motifs have similar hydrophobicity (Table S1), physio-chemical properties, such as the net charge of the franking amino acids, may also affect targeting [23]. A comparison of the transmembrane helix motifs of AtPAP2 and AtPAP9 (orange box in Figure S2) showed a negatively charged glutamate (E) at position a.a. 609 in AtPAP9, but this position (a.a. 620 in AtPAP2) was a positively charged lysine (K) in AtPAP2 (Figure S2). In addition, nine amino acids upstream of the C-terminal motif in AtPAP2 were missing in AtPAP9 (Figure S2). All of these could affect the targeting of AtPAP9 to mitochondria. 

### 2.2. Overexpression Lines of AtPAP9 Did Not Exhibit Growth-Promoting Phenotypes

**Figure 2:**
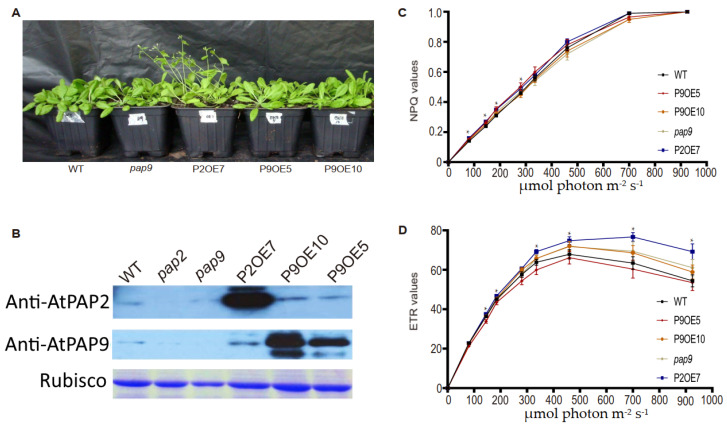
Growth phenotypes of AtPAP9 OE lines. (**A**) Thirty-day-old soil-sown transgenic plants growing under long-day conditions (16 h light at 22 °C/8 h dark at 18 °C). Each black soil pot contained nine individual plants of the same line. Two independent T_3_ AtPAP9 OE lines (P9OE5 and P9OE10) are shown. (**B**) Immunoblot analysis of total leaf protein using anti-AtPAP2 and anti-AtPAP9 antibodies, and gel staining of rubisco as the loading control. (**C**,**D**) Chlorophyll fluorescence analysis. Light-intensity-dependent NPQ (**C**) and ETR (**D**) were plotted. Data for each line are presented as the mean ± SE (*n* > 10 per line). The statistical difference was determined by one-way ANOVA followed by multiple comparisons using the Tukey method. Asterisks indicate that the values of P2OE7 are significantly higher than those in the WT line (* *p* ≤ 0.05). P9OE lines and *pap9* did not exhibit any significant difference in both parameters compared to the WT line. The plants were dark-adapted for 1 h before measurement.

To decipher the role of AtPAP9 in vivo, we generated T_3_ homozygous AtPAP9 overexpression (OE) lines driven by the cauliflower mosaic virus (CaMV) 35S promoter. Two homozygous lines expressing full-length AtPAP9, P9OE5, and P9OE10 (Figure 2A) were selected from multiple independent lines with high AtPAP9 protein expression (Figure 2B). A homozygous T-DNA line of *AtPAP9* (Salk_129905) was obtained from the Arabidopsis Biological Resource Center (ABRC). The T-DNA insertion at the first exon of *AtPAP9* (+465 nucleotide from the ATG start codon) abolished its protein expression (Figure 2B). 

Under long-day (16 h light at 22 °C/8 h dark at 18 °C) growth conditions, the OE lines of AtPAP9 did not display any growth promotion effect compared with wild-type *Arabidopsis thaliana* ecotype Columbia-0 (WT) and T-DNA lines (Figure 2A). Overall, the typical early-bolting and high-seed-yield phenotypes of the AtPAP2 OE line (OE7) were not observed in the OE lines of AtPAP9 (Table 1) [8,19]. The electron transport rate (ETR) and non-photochemical quenching (NPQ) of the AtPAP9 OE and T-DNA lines were similar to the WT line, which correlated with their growth phenotypes (Figure 2C).

### 2.3. AtPAP9 Modulates the Import Rate of [^35^S]-Labeled AtSSU1B into Chloroplasts

**Figure 3:**
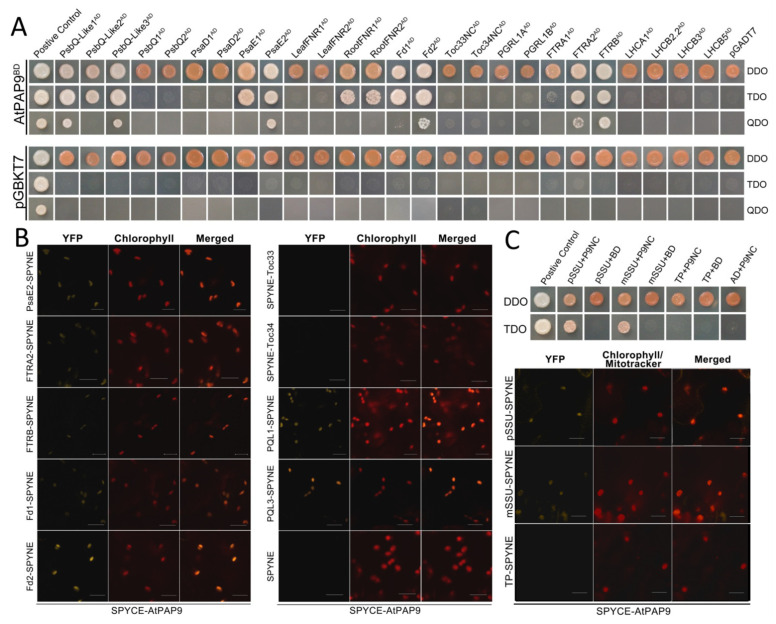
AtPAP9 interacted with a number of chloroplast proteins. (**A**) In Y2H assay, AtPAP9 without its signal peptide and C-terminus (a.a. 21–605, P9NC) was used as the bait and cloned into the pGBKT7 BD vector. It was then co-transformed with the pGADT7 AD vectors carrying the prey. To test for auto-activation, blank pGBKT7 BD vector and pGADT7 AD vector were co-transformed with the bait and prey, respectively. The yeast transformants were diluted 10x and selected on double-dropout (DDO, SD/-Leu/-Trp), triple-dropout (TDO, SD/-Leu/-Trp/-His), and quadruple-dropout (QDO, SD/-Ade/-Leu/-Trp/-His) media. Yeast colonies grown on DDO medium indicated that both bait and prey vectors were successfully transformed into the cells, while yeast colonies grown on TDO and QDO media indicated medium and strong interaction of the bait and the prey in the yeast cells, respectively. pGBKT7-53 and pGADT7-T vectors were used as positive controls. (**B**) BiFC assay was carried out to confirm the interaction between AtPAP9 and the interacting proteins. YFPN-fused chloroplast proteins and YFPC-AtPAP9 were co-expressed in tobacco leaves by syringe injection. Empty SPYNE vectors were used as negative controls. Reconstituted YFP fluorescence was excited at 515 nm and detected with a PMT detector at an emission bandwidth of 530–550 nm (left panel). Chlorophyll autofluorescence was excited at 488 nm and detected with a PMT detector at an emission bandwidth of 650–710 nm (middle panel). An overlay of the YFP signal and the chlorophyll autofluorescence is shown in the right panel. Scale bars = 20 µm. All images were captured at the same gain settings as the corresponding PMT channels. (**C**) AtPAP9 interacted with pSSU (a.a. 1–181) and mSSU (a.a. 42–181) but not with the transit peptide of SSU (TP, a.a. 1–50 of pSSU) in Y2H and BiFC assays.

**Figure 4:**
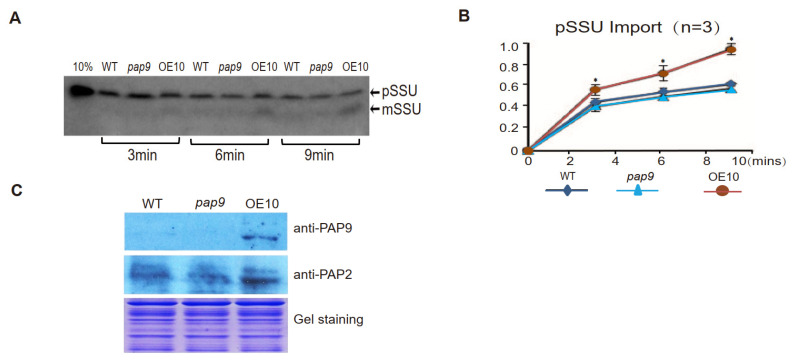
Import assay of [^35^S]-labeled pSSU into chloroplasts. (**A**) Chloroplast import reaction contained chloroplasts equivalent to 16 μg of chlorophyll and 6 μL of wheat germ extract (WGE)-synthesized pSSU; 10% of labeled pSSU added to the import assay was used as a loading control. (**B**) Qualification of import assay in (**A**) by image J. The maximal amount of imported mSSU in the OE10 line at 9 min was taken as 1.0. The import rate of pSSU into OE10 chloroplasts was significantly higher than that of WT and *pap9* chloroplasts (* *p* ≤ 0.05). Statistical difference was determined by one-way ANOVA followed by multiple comparisons using the Tukey method. (**C**) Chloroplasts isolated from WT, *pap9*, and OE10 lines were separated by 12% (*v*/*v*) SDS-PAGE, and Western blotting was carried out using anti-AtPAP2 and anti-AtPAP9 antibodies.

AtPAP2 interacts with the mature protein portion of a small subunit of rubisco (mSSU) and a number of chloroplast proteins, and plays a role in the import of pSSU into chloroplasts [11,19]. To compare the binding specificities of AtPAP2 and AtPAP9, yeast two-hybrid (Y2H) assay (Figure 3A) and bimolecular fluorescence complementation assay (BiFC) (Figure 3B) were carried out. The data of both assays confirmed that AtPAP9, like AtPAP2, can bind to the precursor of SSU (pSSU), mSSU, and a number of chloroplast proteins with the same specificity (Figure 4) [19]. Among the photosystem proteins, AtPAP9 interacted with the proteins located at the acceptor side of PSI, including Fd1, Fd2, root FNR1, root FNR2, FTRA2, FTRB, PsaE1, PsaE2, and three PsbQ-like proteins of the NDH subcomplexes (Figure 3A). In contrast, like AtPAP2, AtPAP9 did not interact with PsbQ1/Q2, PGRL1A/B, or the LHC proteins we tested. We also tested whether AtPAP9 can interact with Toc33 and Toc34, as all these proteins are anchored to the outer membranes of chloroplasts via their C-terminal transmembrane motifs and play a role in chloroplast protein import. Our data showed that they do not interact with each other (Figure 3). 

AtPAP2 on the outer membrane of chloroplasts modulates the import of [^35^S]-pSSU into chloroplasts [11]. Hence, we compared the import efficiency of [^35^S]-pSSU into chloroplasts of the WT, *pap9* (T-DNA), and AtPAP9 OE10 lines. The chloroplasts of the OE10 line imported [^35^S]-pSSU at a significantly higher rate than that of the WT line, while the import rate of [^35^S]-pSSU into *pap9* chloroplasts was similar to that into WT chloroplasts, possibly due to the presence of AtPAP2 on the *pap9* chloroplasts (Figure 4B). Hence, AtPAP9, like AtPAP2, also plays a role in pSSU import into chloroplasts. 

## 3. Discussion

Brassica species tend to possess more than one copy of PAPs with a hydrophobic C-terminal motif, such as *Camelina sativa* (two copies) [24], *Brassica rapa* (four copies), and *Brassica napus* (eight copies). However, most other plant species usually only have one copy of PAP with a hydrophobic C-tail in their genomes, such as the smallest free-living photosynthetic eukaryote (*Ostreococcus tauri*) [8]. The presence of multiple copies of the *AtPAP2*-like gene reflects the ancient polyploidization events in the family Brassicaceae [25]. AtPAP9 is highly expressed in the roots, stems, and senescent leaves but is less expressed in photosynthetically active tissue, such as mature rosette leaves, cauline leaves, and developing siliques (Figure S3). This expression pattern is similar to that of AtPAP2 [8], and hence, they may have evolved by gene duplication in Brassicaceae during evolution. 

**Figure 5:**
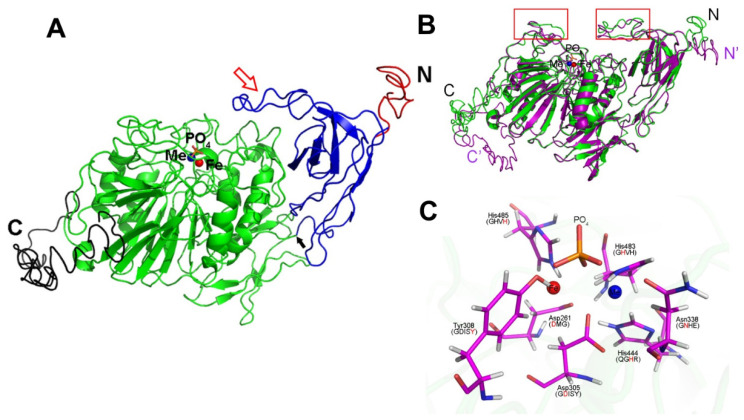
Predicted structure of AtPAP9. (**A**) Different domains of an AtPAP9 monomer unit are shown in different colors (red, transit peptide; blue, FN-III-like domain; green, PAP catalytic domain; black, C-terminal hydrophobic motif; blue sphere, Me (Zn or Mn) atom; red sphere, Fe atom; orange spheres, PO_4_). The flexible loop is indicated by a red arrow, and the hinge connecting the FN-III-like and PAP catalytic domains is indicated by a black arrow. (**B**) The alignment of AtPAP2 (purple) and AtPAP9 (green) is shown as a cartoon by indicating their N- and C-termini, respectively. The metal atoms and phosphate ligands are displayed as spheres. The main different areas are boxed in red frames. (**C**) Active site and bound phosphate of AtPAP9. The Fe and Me atoms are displayed as red and blue spheres, respectively. The phosphate ligands are shown as sticks (red and yellow). The Fe atom is coordinated by amino residues His485, Tyr308, Asp261, and Asp305. The Me atom is coordinated by His444, His 483, Asn338, and Asp305. All the metal-chelating residues are shown as sticks as well by labeling the five conserved PAPs motifs (DMG, GDISY, GNHE, QGHR, and GHVH).

AtPAP2 and AtPAP9 share a 72% amino acid sequence identity (Figure S2). Four functional domains can be identified on the coding sequences of AtPAP2 and AtPAP9—an N-terminal peptide, a fibronectin type III domain (FN-III-like), a phosphatase domain, and a hydrophobic motif at the C-termini (Figure 5A). Three-dimensional (3D) modeling based on the X-ray structure of *Lupines luteus* purple acid phosphatase PPD1 [26] predicted that AtPAP2 and AtPAP9 have highly similar 3D structures (Figure 5B,C). While the putative transit peptides of AtPAP2 and AtPAP9 are highly homologous (Figure S1), the putative transit peptide of AtPAP2 does not play a targeting function [8]. AtPAP2 is a tail-anchored protein dually targeted to chloroplasts and mitochondria via its C-terminal transmembrane motif [8,9]. GFP-targeting experiments demonstrated that the native C-terminal hydrophobic motif of AtPAP9 can only target GFP to chloroplasts (Figure 1). The amino acid differences in the C-tails of AtPAP2 and AtPAP9 after the gene duplication event in the Arabidopsis genome may have affected their ability to target to mitochondria. Y2H and BiFC experiments indicated a similar protein-binding specificity between AtPAP9 and AtPAP2 (Figure 3) [19]. The chloroplast import assay using [^35^S]-labeled AtSSU1B showed that OE of AtPAP9, like AtPAP2, can enhance the import of AtSSU1B into chloroplasts [11]. This is also supported by the 3D modeling of this enzyme in which a similar spatial arrangement between AtPAP2 and AtPAP9 was observed (Figure 5B). 

OE of AtPAP2 on both chloroplasts and mitochondria simultaneously modulates their activities so that the plant cells can generate more ATP and sucrose, thus promoting plant growth [19]. While AtPAP9 shares a 72% sequence identity with AtPAP2, no early-bolting and high-seed-yield phenotypes like those of the AtPAP2 OE7 line were observed in the OE lines of AtPAP9 (Figure 2). This raised the question of whether the function of AtPAP9’s phosphatase domain is different from that of AtPAP2. Y2H assay indicated similar ligand-binding abilities of AtPAP2 and AtPAP9 to a number of chloroplast proteins (Figure 3A), but we cannot rule out the possibility that they differentially interact with some other substrates. Another possible reason for different growth phenotypes of the AtPAP2 and AtPAP9 OE lines (Figure 2) could be the inability of AtPAP9 to target to mitochondria. AtPAP2 plays a role in the import of multiple organellar RNA editing factor 3 (MORF3) into mitochondria [10]. Overexpression of AtPAP2 on mitochondria enhances its capacity to consume reducing equivalents, which could indirectly resupply NADP^+^ in the stroma to accept electrons from the LEF [19]. As AtPAP9 is not targeted to the outer membrane of mitochondria, it is unlikely that its overexpression has any direct impact on mitochondria. 

Another piece of evidence showing that AtPAP9 performs a different role from AtPAP2 is the growth phenotypes of their T-DNA lines. The T-DNA insertion site of *atpap9* (Salk_129905) is located at the first exon (+465 nucleotides from the ATG start codon), and the line can grow like the WT line under normal growth conditions (Figure 2). However, a study reported that T-DNA insertion at the second exon of *AtPAP9* exerts an embryonic lethal effect [21]. In contrast, the *AtPAP2* T-DNA line (*atpap2*, Salk_013567), which also carries a T-DNA insertion at the second exon, does not exhibit an obvious growth difference compared with the WT line [8]. This further supports functional differences between AtPAP2 and AtPAP9. Collectively, AtPAP9 is functionally distinct from AtPAP2, although it may perform a redundant role in assisting the import of some preproteins into chloroplasts. 

Interestingly, the overexpression of AtPAP2 in Arabidopsis, Camelina [27], and potato [28] promotes plant growth and yield, whereas the overexpression of AtPAP9 in Arabidopsis has no growth-promoting effects. This raises a question on the evolutionary relationship between *AtPAP2* and *AtPAP9*. One possibility is that *AtPAP2* is a gain-of-function of *AtPAP9* during gene duplication; the other is that *AtPAP9* evolved later and is a loss-of-function of its paralogous partner *AtPAP2*. Further functional comparison between these OE lines, including characterization of their chloroplasts and mitochondria, will lead to a better understanding of the physiology of these two energy-generating organelles.

## 4. Materials and Methods

### 4.1. Plant Materials and Growth Conditions

The T-DNA insertion mutants of *AtPAP9* (Salk_129905, Col-0) and *AtPAP2* (Salk_129905, Col-0) were obtained from the Arabidopsis Biological Resource Center (ABRC, http://abrc.osu.edu/ (accessed on 1 July 2021)) and were verified as homozygous mutants by genomic PCR and RT-PCR [8]. Arabidopsis seeds were sterilized with 20% (*v*/*v*) bleach for 15 min. The seeds were sown on Murashige and Skoog (MS) agar supplemented with 2% (*w*/*v*) sucrose. To obtain soil-grown plants, 10-day-old seedlings were transferred to soil, where they were allowed to grow under long-day (16 h light at 22 °C/8 h dark at 18 °C) growth conditions.

### 4.2. Isolation of Chloroplasts from Arabidopsis Leaves

Arabidopsis seeds were sown and grown on Murashige and Skoog (MS) agar supplemented with 2% (*w*/*v*) sucrose under long-day (16 h light at 22 °C/8 h dark at 18 °C) growth conditions. Chloroplasts were isolated from the leaves of 14-day-old Arabidopsis plants using the cellulase method [29,30].

### 4.3. Generation of Overexpression Lines of AtPAP9

The full-length coding sequence (CDS) of *AtPAP9* (At2g03450) cDNA was amplified with *Pfx* DNA polymerase (Invitrogen) using primers P9-F and P9-R (Table S2). The 1956 bp PCR product was cloned into the plant transformation pCXSN vector via TA cloning [31]. The expression of *AtPAP9* was driven by the CaMV 35S promoter (pCXSN-CaMV35: AtPAP9). Plant transformation was performed, as previously described [32]. Seeds were screened on MS agar plates containing 30 μg/mL of hygromycin. T_1_ seedlings were transferred to soil and subjected to Western blotting analyses. T_2_ seedlings that followed a segregation pattern (resistant:susceptible = 3:1) were selected and transferred to soil. Homozygous T_3_ lines were subsequently identified for further analysis.

### 4.4. Western Blotting Analysis

The antibody of AtPAP9 was produced by PolyExpress™ Antibody Services (GenScript, Nanjing, China). The AtPAP9-specific peptide (a.a. 450–468, YTTSRKIRDAAIREKMIEH) designed using the OptimumAntigen design tool (GenScript, Nanjing, China) was used to immunize rabbits (Figure S2). The antibodies (anti-AtPAP9) were affinity-purified by the peptides. Anti-PAP2 antibodies were generated, as previously described [8]. Horseradish peroxidase (HRP)-labeled secondary antibodies were used, and protein bands were visualized using the WesternBright^TM^ Quantum Western blotting detecting kit (Advansta, San Jose, LA, USA).

### 4.5. Subcellular Localization Analysis by GFP

Arabidopsis mesophyll protoplast preparation and transfection were carried out, as previously described [33]. Briefly, 0.1 mL (10^5^) of protoplasts isolated from 4-week-old rosettes were transfected with 8 μg of plasmids and cultured for 16 h for protein expression. Plasmids for transient expression were extracted using a Hipure EF Plasmid Kit (Magen, Shenzhen, China). The C-terminal motif of AtPAP9 (a.a. 606–651) was amplified with the primer pairs P9C2GFP-F and P9CGFP-R (Table S2) and cloned into a GFP vector (pBI221) to generate the plasmid GFP-P9C. GFP-P2C1 (a.a. 615–656 of AtPAP2) was used as a control for comparison [9]. Chlorophyll and mitochondrial F1-RFP were used as organelle markers [34].

### 4.6. Yeast Two-Hybrid Assay and Bimolecular Fluorescence Complementation Analysis

The Matchmaker™ Gold Yeast Two-Hybrid system was used (Tanaka, Kasatsu, Japan). The PCR product of AtPAP9 (a.a. 21–605), lacking its N-terminal peptide and C-terminal transmembrane motif, was cloned to the C-terminus of the GAL4 DNA-binding domain (BD) of the pGBKT7 vector. The pGADT7 vectors carrying potential interacting partners were prepared, as previously described [11,19]. pGBKT7-53 and pGADT7-T vectors were used as positive controls. 3-AT was not used in the Y2H assay. For BiFC assay, the full-length cDNA of AtPAP9 was cloned into the pSPYNE vector, while the pSPYCE vectors carrying interacting partners were prepared, as previously described [11,19]. Y2H and BiFC assays were carried out, as previously described [35].

### 4.7. Chloroplast Import Assays

Import-competent chloroplasts were isolated from 14-day-old Arabidopsis seedlings growing on MS agar plates, as previously described [29]. The [^35^S]-labeled AtSSU1B (At5g38430) precursor protein was translated in vitro using the TNT^®^-Coupled Wheat Germ Extract (WGE) System (Promega Corporation) [11]. The post-ribosome supernatant was prepared from freshly translated [^35^S]-AtSSU1B in WGE (48,100× *g*, 20 min, 4 °C). The chloroplast import reaction was performed under white light at 25 °C in import buffer containing 50 mM HEPES/KOH, 3 mM MgSO_4_, 0.33 M sorbitol, 20 mM K-gluconate, 10 mM NaHCO_3_, 0.2% (*w*/*v*) BSA, 5 mM Mg-ATP, and 10 mM methionine at pH 8.0 [11,36]. The import reaction was terminated with an equal volume of stop buffer (50 mM HEPES/KOH, 0.33 M sorbitol, and 50 mM EDTA). 

### 4.8. Chlorophyll Fluorescence Measurements

The chlorophyll fluorescence levels of Arabidopsis leaves were monitored with IMAGING-PAM M-Series Maxi Version (WALZ, Effeltrich, Germany). The plants were dark-adapted for at least 1 h before measurement. After the maximum (F_m_) and initial (F_o_) fluorescence values were determined with a delay of 40 s, the plants were illuminated at the following light intensities: 0, 81, 145, 186, 281, 335, 461, 701, and 926 µmol photon m^−2^ s^−1^. The duration of illumination at individual light intensities was 3 min. A saturation pulse was applied at the end of each 3 min illumination.

### 4.9. 3D Computer Modeling

The structure of 3ZK4 (purple acid phosphatase PPD1 isolated from *Lupines luteus*) was previously found to be the best-fitting structure of AtPAP2 and AtPAP9 in the NCBI (https://blast.ncbi.nlm.nih.gov (accessed on 1 July 2021)). Therefore, 3ZK4 was used as a reference to align the predicted structure of AtPAP2 and AtPAP9 with I-TASSER (http://zhanglab.ccmb.med.umich.edu/I-TASSER/ (accessed on 1 July 2021)). PyMOL v1.3r1 (released in 2010) was used for subsequent editing.

## Figures and Tables

**Table 1:** Seed yield at maturity.

Lines	Siliques per Plant	Seed Yield (g per Plant)
WT	367 ± 50 ^a^	0.203 ± 0.047 ^a^
*PAP9*	353 ± 34 ^a^	0.198 ± 0.062 ^a^
P2OE7	577 ± 81 ^b^	0.313 ± 0.093 ^b^
P9OE5	350 ± 36 ^a^	0.209 ± 0.077 ^a^
P9OE10	358 ± 47 ^a^	0.230 ± 0.061 ^a^

*n* = 12. Statistically significant difference was determined by one-way ANOVA followed by multiple comparisons using the Tukey method using GraphPad 7.04. Within each column, the values marked by different letters (a, b) are significantly different (*p* < 0.05).

## Data Availability

The data supporting the findings of this study are available within the article and its Supplementary Materials.

## Supplementary Materials

The following are available online at https://www.mdpi.com/article/10.3390/ijms22147243/s1.
