# Supplementary material for: Overlapping Functions of the Paralogous Proteins AtPAP2 and AtPAP9 in Arabidopsis thaliana
Source: Int J Mol Sci. 2021 Jul 6;22(14):7243. doi: 10.3390/ijms22147243 (PMC8303434; doi:10.3390/ijms22147243)
Supplement: Supplementary file 1 [file ijms-22-07243-s001.zip › ijms-1240359-supplementary.pdf]

A

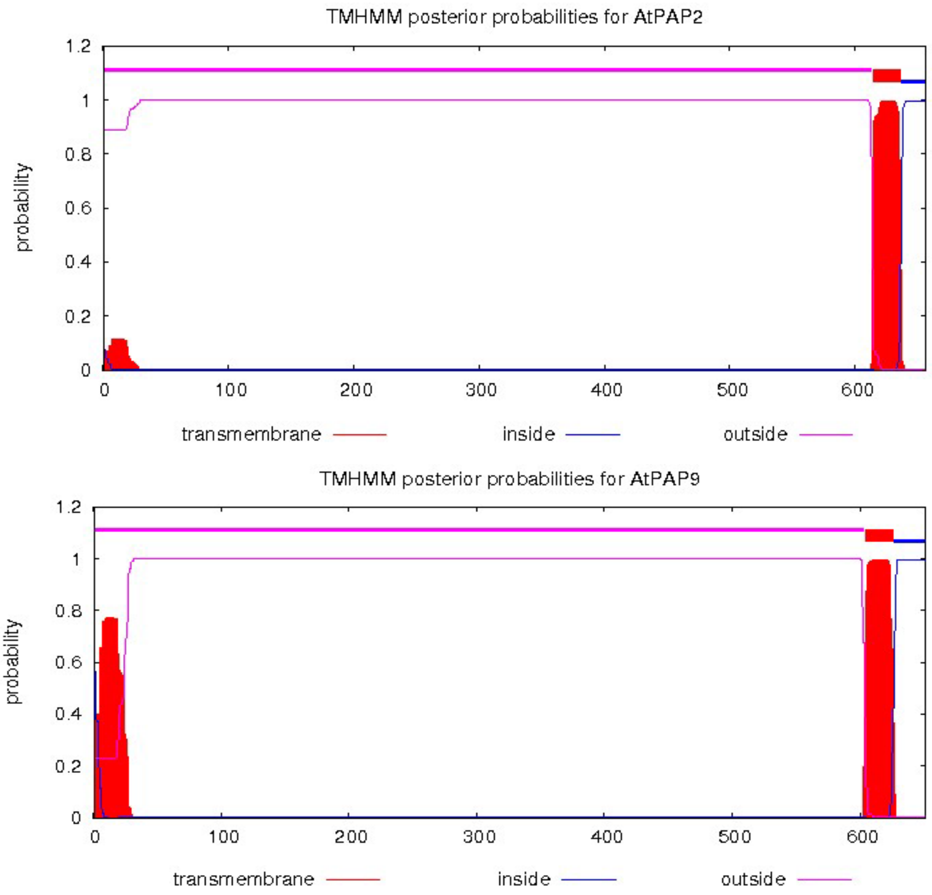

B

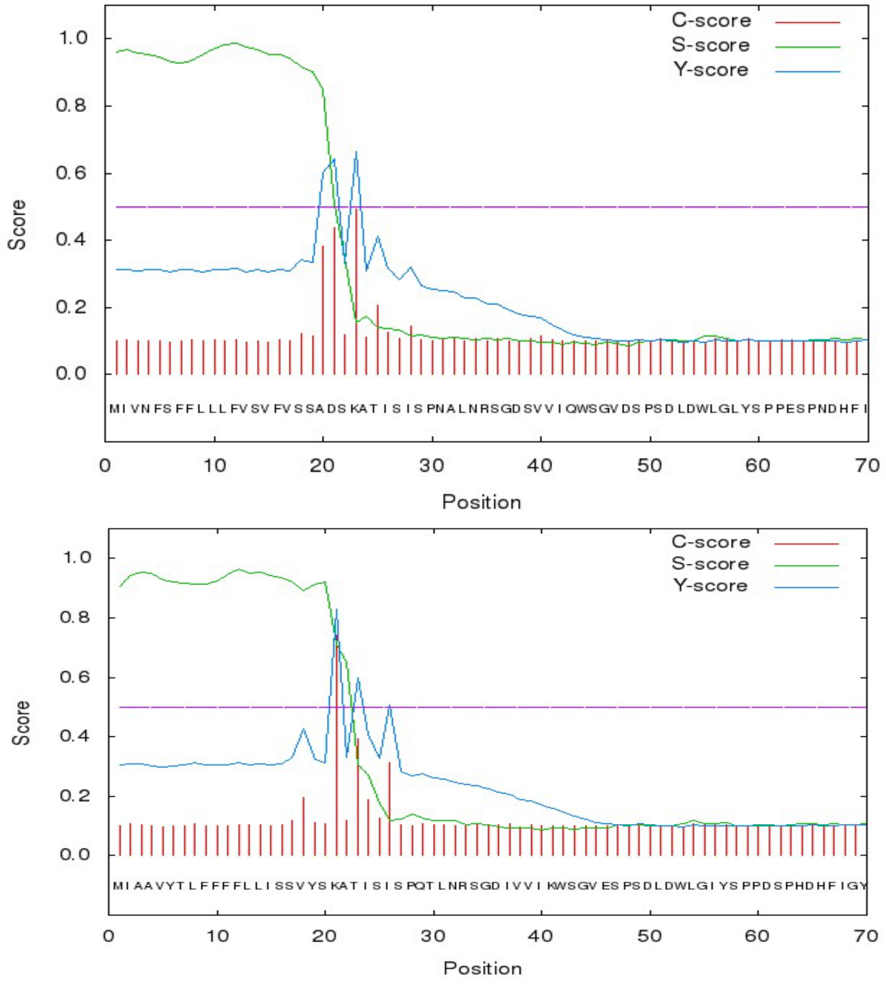

FIGURE S1. Transmembrane motif and signal peptide prediction. (A) TMHMM Server v. 2.0 (http://www.cbs.dtu.dk/services/TMHMM-2.0/) was used to predict the presence of transmembrane motifs (red colored dash) on the protein sequences. **(B)** Signal peptides of AtPAP2 (top) and AtPAP9 (bottom) were predicted by SignalP 4.1 server using the defalt D-cutoff value (0.50, purple line). Raw cleavage site score (C-score), Signal peptide score (S-score), combined cleavage site score (Y-score) were shown (http://www.cbs.dtu.dk/services/SignalP-4.1/ output.php).

Figure S2

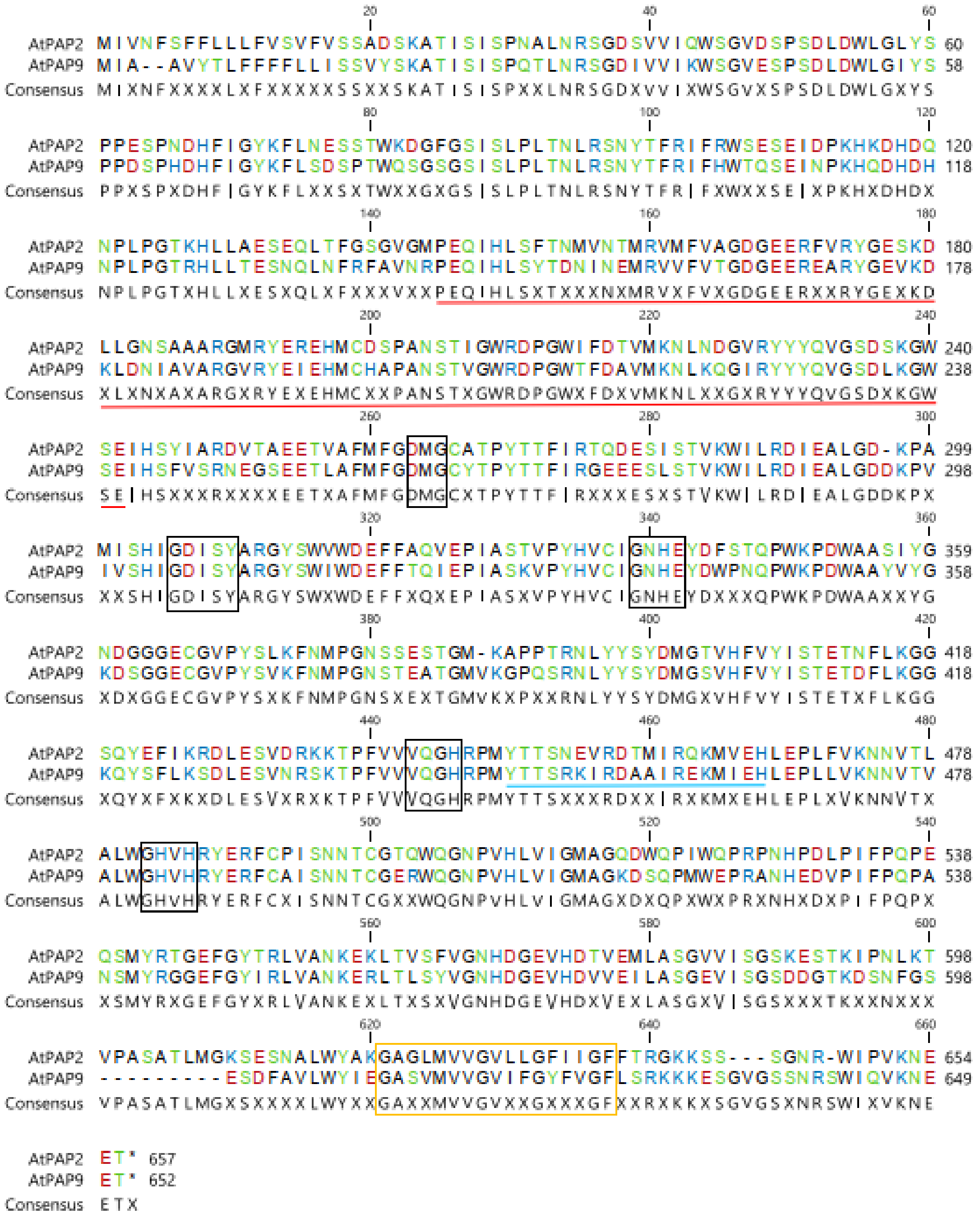

FIGURE S2. Amino acid sequence alignment of AtPAP2 and AtPAP9. The a.a. sequences were aligned by the CLC Sequence Viewer 6 software. The five conserved amino acids motifs were indicated by black boxes. The transmembrane helix (604 - 626 a. a. of AtPAP9) at the C-terminus was indicated by the orange box (TMHMM 2.0.). The fibronectin-like domain was underlined in red. AtPAP9-specific peptide used as antigen was underlined in blue.

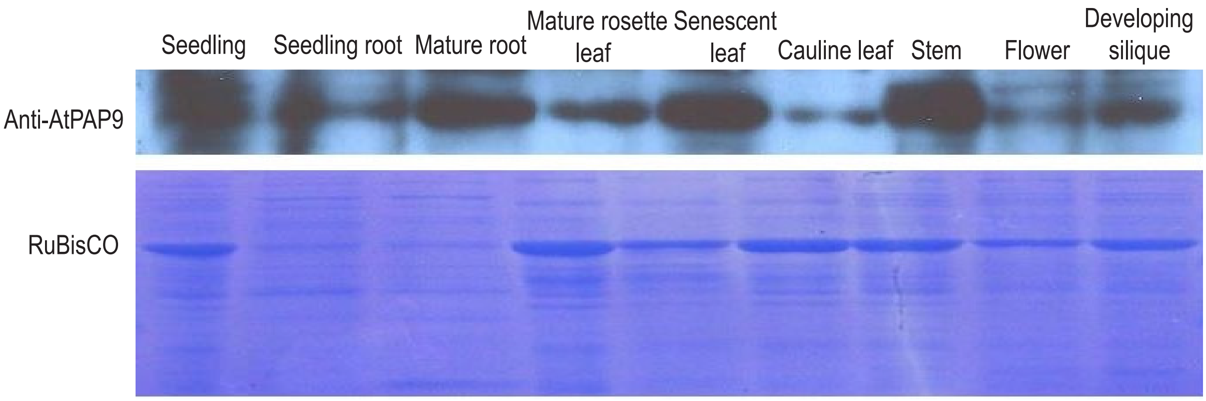

FIGURE S3. **Immunoblotting of AtPAP9 in WT *Arabidopsis thaliana*.** The seedling and seedling root samples were harvested from 10-d-old plants growing on MS agar plates. The mature rosette leaf, cauline leaf, stem, flower, and developing silique were harvested from 32-d-old plants growing on soil under long day light scheme (16hr light/8hr dark). The senescent rosette leaf was harvested from 35-d-old plants. For each sample, 20 microgram of protein were loaded. A SDS-PAGE gel stained with coomassie brilliant blue is shown in the bottom panel. The thick bands in green tissues are Rubisco.

TABLE S1. C-terminal hydrophobic motifs of AtPAP2, AtPAP9 and pre-protein receptors of TOC and TOM.

Name	AGI	Sequence	Hydrophobicity K&D	
			H	μH
AtPAP2	At1g13900	LMGKSESNALWYAKGAGLMVVGVLLGFIIGFFTRGKKSSSGNRWIPVKNEET*	2.39	0.66
AtPAP9	At2g03450	LWYIEGASVMVVGVIFGYFVGFLSRKKKESGVGSSNRSWIQVKNEET*	2.09	0.63
**Plastids**				
AtToc33	At1g02280	KKMVDGSYSDDKGKKLIPLIIGAQYLIVKMIQGAIRNDIKTSGKPL*	1.51	1.32
AtToc34	At5g05000	KLVEGPNPNERGKKLIPLMFAFQYLLVMKPLVRAIKSDVSRESKPAWELR*	1.73	0.17
**Mitochondria**				
AtTom20-1	At3g27070	KNKKSSDEKYIVMGWVILAIGVVACISFRKLR*	2.37	0.56
AtTom20-2	At1g27390	KKRNTEFTYDVCGWIILACGIVAWVGMAKSLGPPPPAR*	2.17	0.56
AtTom20-3	At3g27080	NKKSSDAKYDAMGWVILAIGVVAWISFAKANVPVSPPR*	2.13	0.43
AtTom20-4	At5g40930	QKKTSEFKYDVFGWVILASYVVAWISFANSQTPVSRQ*	1.97	0.49

The mean hydrophobicities were calculated by hydromCalc (http://www.bbcm.univ.trieste.it/~tossi/hydroCalc/hydromCalc.html#hiscale) using Kyte and Doolittle (K&D) hydrophobicity scale. H and μH were the total sum of all residue hydrophobicity indices and the vectorial sum of all the hydrophobicity indices divided by the number of residues, respectively. Hydrophobic motifs were underlined; blue color represent negatively charged amino acid and red color represented positively charged amino acid; * denoted stop codon.

TABLE S2. Primers used in this study.

Vector (Purpose)	Gene Name (AGI code)	Primer Sequence (5'-3')[1]
pCXSN (Plant transformation)	1-651aaAtPAP9 (AT2G03450)	P9-F: AAAACTCGAGATGATCGCCGCCGTTTAC<br>P9CGFP-R: GCTTGAGCTCTCATGTCTCCTCGTTTTTC
pBI221-GFP (Sub-cellular localization)	606-651aaP9C (AT2G03450)	P9C2GFP-F: TGCAACTAGTTGGTACATTGAAGGAGCAAGT-<br>-GTGATGGTTGTGGGAGTGATTTTTGGGTACTTTGTCGGT<br>P9CGFP-R: GCTTGAGCTCTCATGTCTCCTCGTTTTTC
pGBKT7 (Yeast two-hybrid)	25-613a.a. AtPAP2 (AT1G13900)	F: TTCTCATATGACCATTTCAATTTCCCC<br>R: GCATGTCGACCAGCATTAGATTCTGATTTTC
pGBKT7 (Yeast two-hybrid)	21-605a.a. AtPAP9 (AT2G03450)	F: TAATCCATGGTTAAAGCCACGATTTCAATCT<br>R: CGATGTCGACTCACAAGACTGCAAAGTCAGAT
pGADT7 (Yeast two-hybrid)	1-50a.a. SSU1B (AT5G38430)	F: ATTACATATGGCTTCCTCTATGCTCTCCT<br>R: ATTACTCGAGCCCATTGCTTGTGATGGAAG
pGADT7 (Yeast two-hybrid)	42-181a.a. SSU1B (AT5G38430)	F: ATTCCATATGGACATTACTTCCATCACAAGC<br>R: ATTACTCGAGAGCATCAGTGAAGCTTGGGG
SPYCE (BiFC)	AtPAP2 (AT1G13900)	F: ATCGACTAGTATGATCGTTAATTTCTCTTTCTTC<br>R: GAATACTAGTTTATGTCTCCTCGTTCTTGACTG
SPYCE (BiFC)	AtPAP9 (AT2G03450)	F: TAATACTAGTATGATCGCCGCCGTTTACAC<br>R: TAATACTAGTTCATGTCTCCTCGTTTTTCACTTGG
SPYCE (BiFC)	Multiple cloning site	F: ATTAGAGCTCGTTAACCGGGCTCAGGCCT<br>R: ATTAGAGCTCCCCGGGAGCGGTACCCTC
SPYCE (BiFC)	YFP^C	F: TAACTCTAGAATGTACCCATACGATGTTCCAG<br>R: ATTAGAGCTCCTTGTACAGCTCGTCCATG

[1]Restriction enzyme sites are underlined.
